# Supplementary material for: Mycotoxins from Tomato Pathogenic Alternaria alternata and Their Combined Cytotoxic Effects on Human Cell Lines and Male Albino Rats
Source: J Fungi (Basel). 2023 Feb 21;9(3):282. doi: 10.3390/jof9030282 (PMC10054162; doi:10.3390/jof9030282)
Supplement: Supplementary file 1 [file jof-09-00282-s001.zip › jof-2194635-supplementary.pdf]

Table S1. Reference sequences retrieved from GenBank database used for phylogenetic analysis.

**Species Name**	**Isolate Number**	**Host**	Location	GenBank Accession Numbers		
				rpb2	*gapdh*	*Alt-a1*
Alternaria alstroemeriae	**CBS 118808**	***Alstroemeria* sp.**	USA	KP124764	KP124153	KP123845
A. alternantherae	CBS 124392	*Solanum melongena*	China	KC584374	KC584096	KP123846
A. alternata	CBS 916.96^T	*Arachis hypogeae*	India	KC584375	AY278808	KC584375
A. alternata	CBS 119399^T	*Minneola tangelo*	USA	KP124829	JQ646328	KP123910
A. alternata	CBS 119543^T	*Citrus paradisi*	USA	KP124831	KP124215	KP123911
A. alternata	CBS 918.96	*Dianthus chinensis*	UK	KC584435	AY278809	AY563302
A. alternata	CBS 103.33^T	Soil	Egypt	KP124770	KP124159	KP123852
A. alternata	CBS 102.47	*Citrus sinensis*	USA	KP124773	KP124161	KP123855
A. alternata	**ALT2265**	***Solanum lycopersicum***	**Egypt**	**OP893862**	**OP893842**	**OP893822**
A. alternata	**ALT2262**	***Solanum lycopersicum***	**Egypt**	**OP893863**	**OP893843**	**OP893823**
A. alternata	**ALT2261**	***Solanum lycopersicum***	**Egypt**	**OP893864**	**OP893844**	**OP893824**
A. alternata	**ALT2258**	***Solanum lycopersicum***	**Egypt**	**OP893865**	**OP893845**	**OP893825**
A. alternata	**ALT2257**	***Solanum lycopersicum***	**Egypt**	**OP893866**	**OP893846**	**OP893826**
A. alternata	**ALT2255**	***Solanum lycopersicum***	**Egypt**	**OP893867**	**OP893847**	**OP893827**
A. alternata	**ALT2254**	***Solanum lycopersicum***	**Egypt**	**OP893868**	**OP893848**	**OP893828**
A. alternata	**ALT2246**	***Solanum lycopersicum***	**Egypt**	**OP893869**	**OP893849**	**OP893829**
A. alternata	**ALT2244**	***Solanum lycopersicum***	**Egypt**	**OP893870**	**OP893850**	**OP893830**
A. alternata	**ALT2242**	***Solanum lycopersicum***	**Egypt**	**OP893871**	**OP893851**	**OP893831**
A. alternata	**ALT2236**	***Solanum lycopersicum***	**Egypt**	**OP893872**	**OP893852**	**OP893832**
A. alternata	**ALT2232**	***Solanum lycopersicum***	**Egypt**	**OP893873**	**OP893853**	**OP893833**
A. alternata	**ALT2230**	***Solanum lycopersicum***	**Egypt**	**OP893874**	**OP893854**	**OP893834**
A. alternata	**ALT2224**	***Solanum lycopersicum***	**Egypt**	**OP893875**	**OP893855**	**OP893835**
A. alternata	**ALT2223**	***Solanum lycopersicum***	**Egypt**	**OP893876**	**OP893856**	**OP893836**
A. alternata	**ALT2215**	***Solanum lycopersicum***	**Egypt**	**OP893877**	**OP893857**	**OP893837**
A. alternata	**ALT2213**	***Solanum lycopersicum***	**Egypt**	**OP893878**	**OP893858**	**OP893838**
A. alternata	**ALT2210**	***Solanum lycopersicum***	**Egypt**	**OP893879**	**OP893859**	**OP893839**
A. alternata	**ALT2208**	***Solanum lycopersicum***	**Egypt**	**OP893880**	**OP893860**	**OP893840**
A. alternata	**ALT2203**	***Solanum lycopersicum***	**Egypt**	**OP893881**	**OP893861**	**OP893841**
Alternaria arborescens	CBS 101.13^T	Peat soil	Switzerland	KP124862	KP124244	KP123940
Alternaria arborescens	CBS 119544^T	*Avena sativa*	New Zealand	KP124878	KP125186	KP123955
Alternaria betae-kenyensis	CBS 118810	*Beta vulgaris var.cicla*	Kenya	JQ905180	JQ905161	JQ905104
Alternaria burnsii	CBS 107.38	*Cuminum cyminum*	India	JQ646457	JQ646305	JQ646388
Alternaria burnsii	CBS 118816	*Rhizophora mucronata*	India	KP124892	KP124273	KP123970
Alternaria burnsii	CBS 118817	*Tinospora cordifolia*	India	KP124893	KP124275	KP123972
Alternaria eichhorniae	CBS 489.92 ^{T}	*Eichhornia crassipes*	India	KP124895	KP124276	KP123973
Alternaria gaisen	CBS632.93	*Pyrus pyrifolia*	Japan	KC584399	KC584116	KP123974
Alternaria gaisen	CBS 118488	*Pyrus pyrifolia*	Japan	KP124897	KP124278	KP123975
Alternaria gossypina	CBS 104.32	*Gossypium* sp.	Zimbabwe	KP124900	JQ646312	JQ646395
Alternaria gossypina	CBS 107.36	soil	Indonesia	KP124901	JQ646310	JQ646393
Alternaria iridiaustralis	CBS 118486^T	*Iris* sp.	Australia	KP124905	KP124284	KP123981
Alternaria iridiaustralis	CBS 118487	*Iris* sp.	Australia	KP124906	KP124285	KP123982

Alternaria jacinthicola	CBS 133751[T]	*Arachis hypogaea*	Mauritius	KP124908	KP124287	KP123984
Alternaria longipes	CBS 540.94	*Nicotiana tabacum*	USA	KC584409	AY278811	AY563304
Alternaria longipes	CBS 121333	*Nicotiana tabacum*	USA	KP124914	KP124293	KP123990
Alternaria tomato	CBS 103.30	*Solanum lycopersicum*	Unknown	KP124915	KP124294	KP123991
Alternaria tomato	CBS 114.35	*Solanum lycopersicum*	Unknown	KP124916	KP124295	KP123992

The ex-type cultures are indicated with superscript (T), and the isolates obtained in this study are boldfaced.

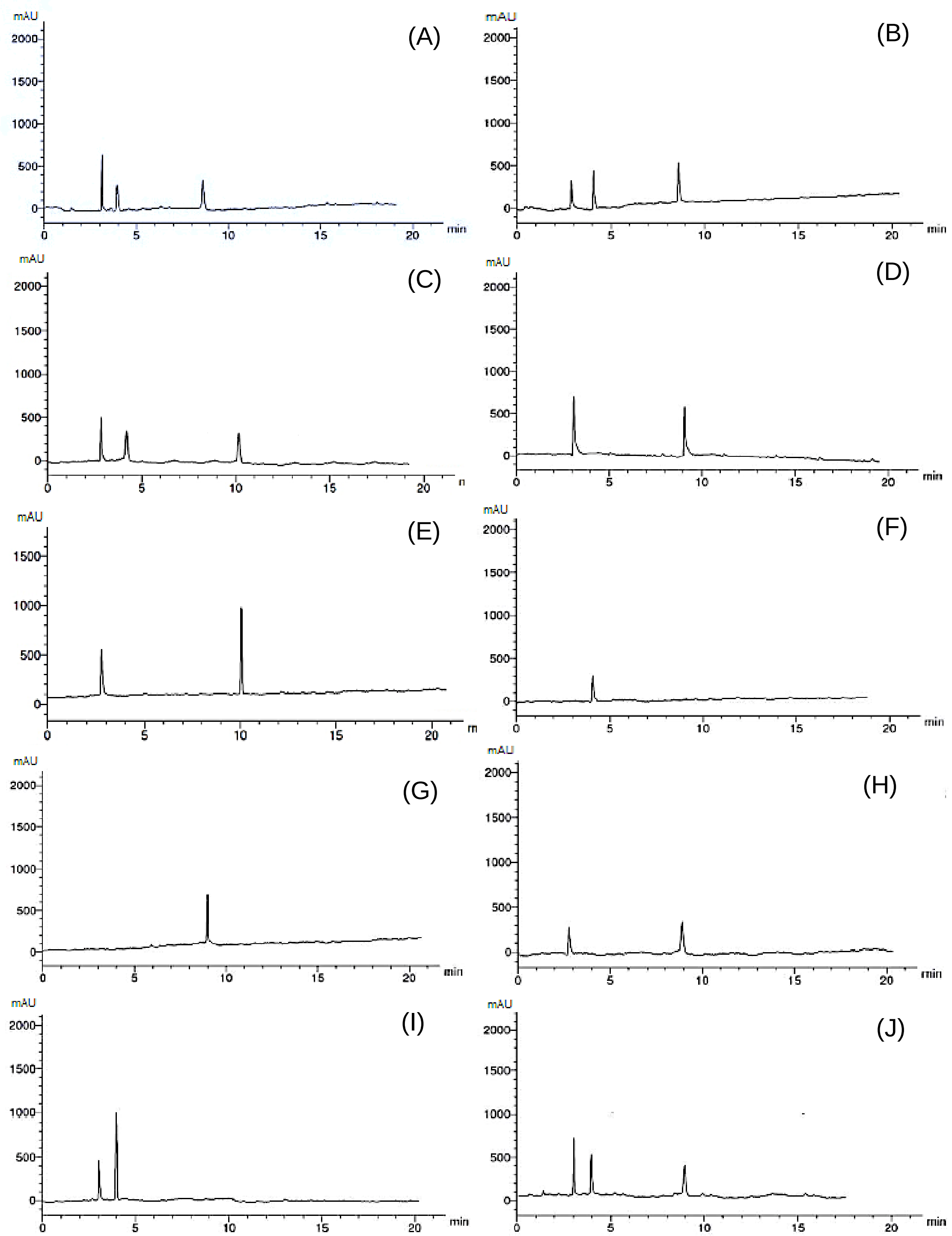

Figure S1. HPLC chromatograms of Altenuene (ALT), Alternariol (AOH), Tenuazonic Acid (TeA) and Altenuisol (AS) produced by *A. alternata* isolates: ALT 2261(A); ALT 2258 (B); ALT 2265 (C); ALT 2210 (D); ALT 2224 (E); ALT 2244 (F); ALT 2254 (G); ALT 2232 (H); ALT 2257 (I); ALT 2215 (J).
